# Fatal intoxication caused by conventional-dose methotrexate in an elderly patient with end-stage renal disease: A cautionary case report

**DOI:** 10.1097/MD.0000000000050331

**Published:** 2026-08-21

**Authors:** Qian He, Xu Huang

**Affiliations:** a Department of Emergency, The Second Affiliated Hospital of Chongqing Medical University, Chongqing, People’s Republic of China; b Department of Emergency, The First Affiliated Hospital of Chongqing Medical University, Chongqing, People’s Republic of China.

**Keywords:** case report, end-stage renal disease, intoxication, methotrexate

## Abstract

**Rationale:**

Methotrexate (MTX) is a first-line agent for rheumatoid arthritis, and impaired renal excretion drastically elevates the risk of fatal drug accumulation. End-stage renal disease (ESRD) patients face an extreme toxicity risk even under conventional low-dose MTX, yet related fatal cases remain underreported. This case highlights critical clinical warnings for rheumatologists and emergency physicians.

**Patient concerns:**

A 71-year-old male with a 5-year history of rheumatoid arthritis and 1-year untreated chronic kidney disease stage 5 received weekly 10 mg subcutaneous MTX for 3 consecutive weeks without folic acid supplementation or regular laboratory monitoring. One day after his latest injection, he developed persistent nausea, vomiting, extensive oral ulcers, a skin rash, and drowsiness.

**Diagnoses:**

Severe MTX intoxication complicated by profound bone marrow suppression, septic shock, and multiple organ dysfunction syndrome. Persistently elevated serum MTX confirmed impaired renal drug clearance in ESRD.

**Interventions:**

Immediate MTX discontinuation, leucovorin calcium rescue, continuous renal replacement therapy, broad-spectrum anti-infection treatment, blood product transfusion, and invasive mechanical ventilation for progressive circulatory and respiratory failure.

**Outcomes:**

Serial serum MTX remained elevated 6 days after the final dose. Despite full intensive supportive care, the patient died of refractory septic shock and irreversible multiorgan failure.

**Lessons:**

Conventional-dose MTX is contraindicated for ESRD patients. Mandatory pre-prescription renal function assessment, regular laboratory monitoring, and folate supplementation are essential to prevent life-threatening MTX toxicity in high-risk elderly populations.

## 1. Introduction

Methotrexate (MTX) is a first-line disease-modifying antirheumatic drug for rheumatoid arthritis (RA) that exerts its pharmacological effects by antagonizing folate metabolism.^[1]^ While generally safe when used under proper monitoring, it is associated with dose-dependent toxicities, including bone marrow suppression, mucosal damage, and liver and kidney injury, which can be fatal in severe cases.^[2]^ Advanced age, renal impairment, hypoalbuminemia, administration route, dehydration, concomitant drugs, infection, and folate deficiency are all high-risk factors for MTX toxicity, among which advanced renal dysfunction confers the highest risk.^[3]^ Approximately 80% of MTX is excreted renally in its unchanged form, making impaired renal function a key risk factor for drug accumulation.^[4]^ Early clinical symptoms of toxicity are insidious. As the condition progresses, pancytopenia, septic shock, and even multiorgan failure may rapidly develop. Diagnosis and treatment are often delayed in elderly patients and those with chronic kidney disease (CKD) due to the underestimation of the risk.^[5]^

The management of severe MTX toxicity is challenging. Once the condition progresses to severe bone marrow suppression and multiorgan damage, the patient’s prognosis is generally poor.^[6,7]^ Of note, current Kidney Disease: Improving Global Outcomes and European Alliance of Associations for Rheumatology guidelines contraindicate standard low-dose MTX for CKD stage 5 patients because of markedly elevated accumulation and fatal toxicity risk.^[8,9]^

This report describes a case of a geriatric patient with end-stage renal disease (ESRD) who developed fatal MTX toxicity following routine low-dose subcutaneous MTX administration without dose adjustment or laboratory monitoring. Unlike previously reported low-dose MTX toxicities occurring in patients with non-CKD stage 5 and favorable outcomes, this patient with end-stage kidney disease died despite treatment. By detailing the complete clinical course, we aim to provide guidance for the safe clinical use of this medication.

## 2. Case presentation

### 2.1. Ethical and consent statement

This case report was conducted in accordance with the Declaration of Helsinki. The need for ethical approval was waived by the Medical Research Ethics Committee of the First Affiliated Hospital of Chongqing Medical University because this is a retrospective case report of a single patient. Written informed consent was obtained from the patient’s authorized relative for publication of this report and any accompanying images.

### 2.2. Patient information

A 71-year-old male patient was transferred from another hospital to our intensive care unit due to symptoms of nausea, vomiting, and oral mucosal ulcers that had persisted for 2 days. The patient had a 5-year history of RA and 1-year CKD stage 5, without regular dialysis before admission. For the 3 weeks prior to presentation, the patient had been receiving weekly subcutaneous injections of 10 mg of MTX for RA. During this period, he did not take folic acid or any medications that might affect MTX clearance. Routine monitoring of serum MTX levels, complete blood count, or renal function was not performed. One day after his most recent dose, the patient presented with persistent nausea and vomiting, accompanied by multiple oral mucosal ulcers and a scattered rash on the face and chest, prompting his visit to the clinic.

### 2.3. Clinical findings

**Figure 1.**
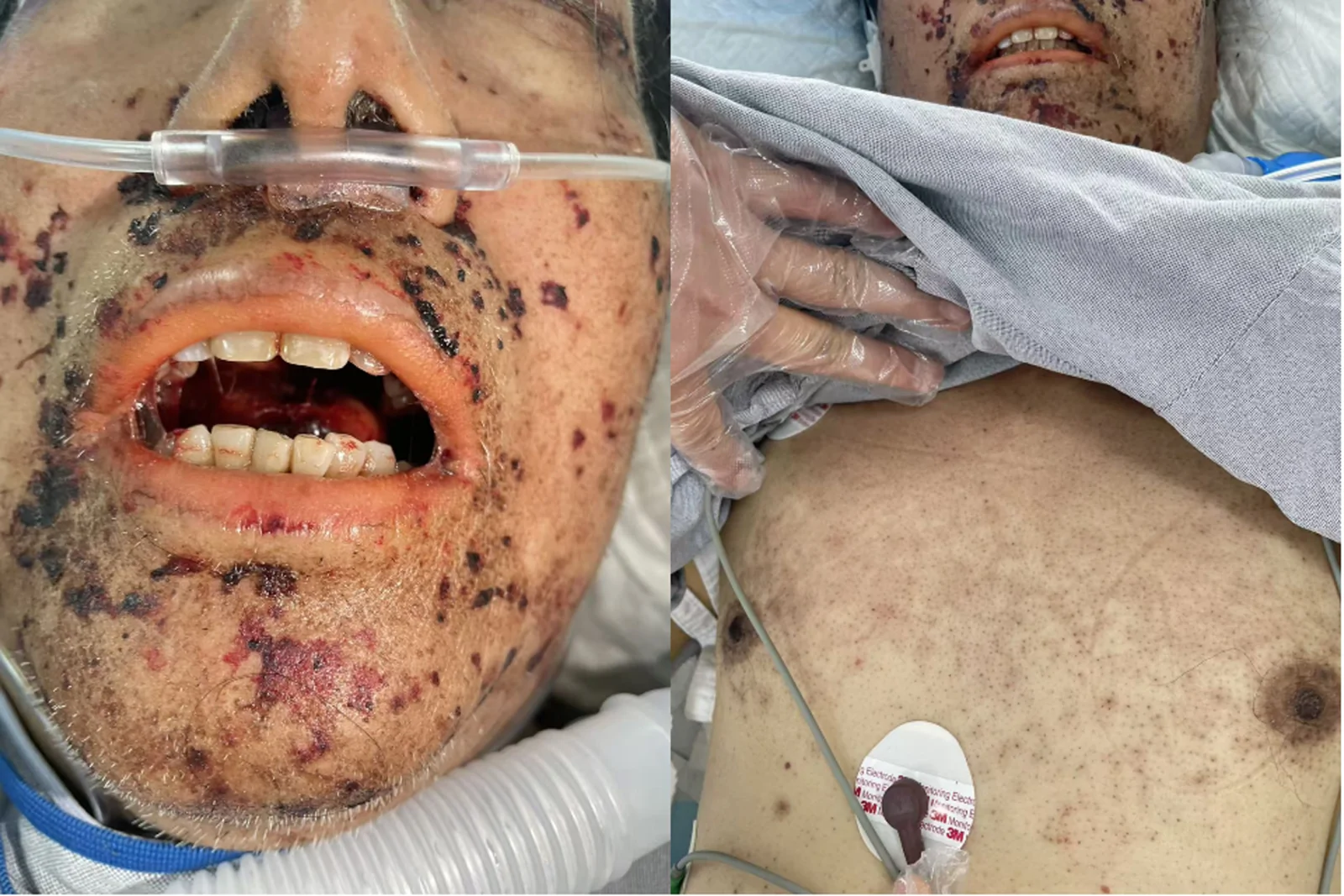
Clinical photographs of the patient at presentation. The left panel shows extensive oral mucosal ulcerations, hemorrhagic crusts, and facial petechiae. The right panel demonstrates a widespread petechial and purpuric rash on the trunk.

**Figure 2.**
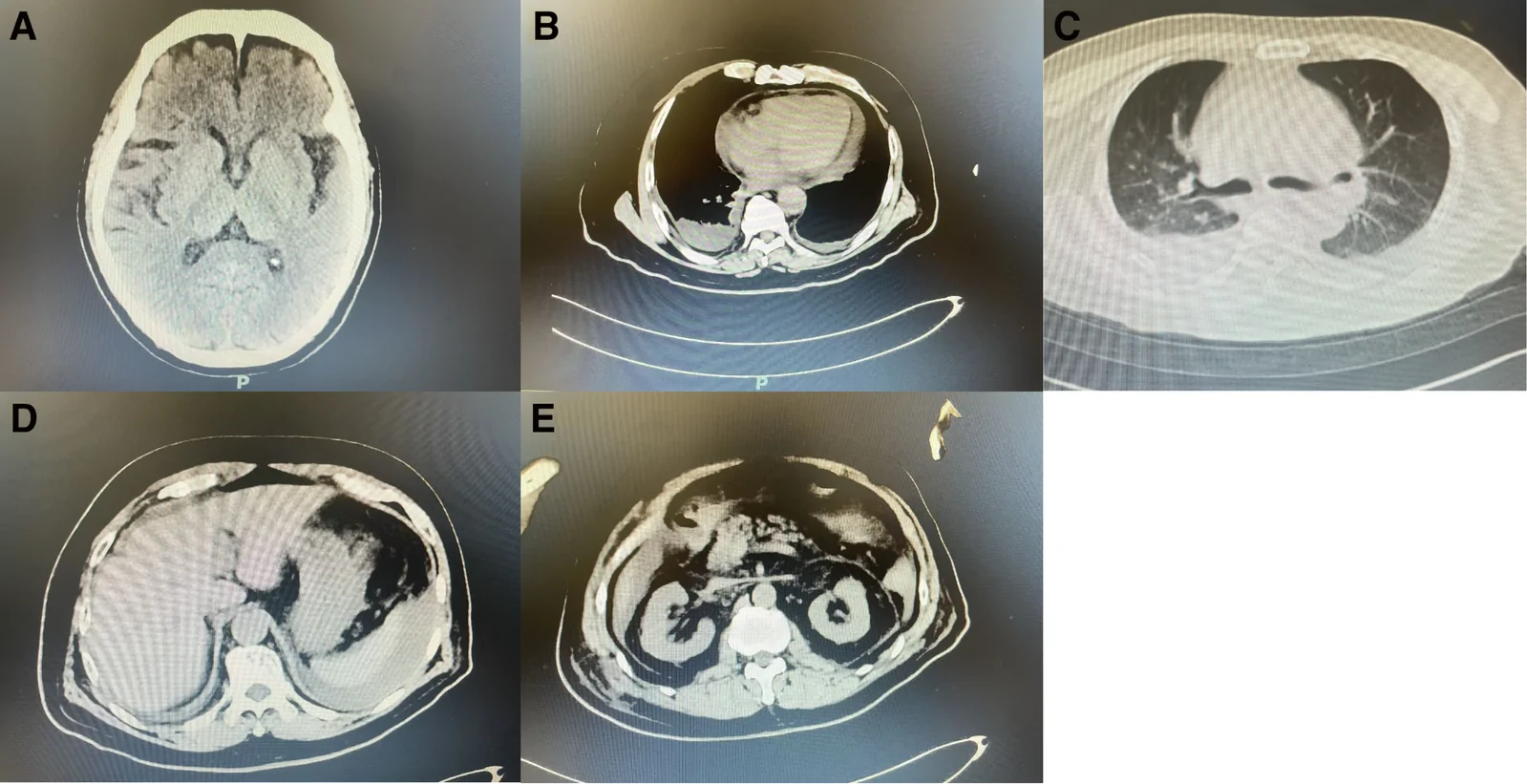
Representative CT scans. (A) Brain CT showing no acute intracranial abnormalities; (B and C) chest CT revealing small bilateral pleural effusions and no focal consolidation; (D and E) abdominopelvic CT showing no significant ascites or organ abnormalities. CT = computed tomography.

Upon admission to our hospital, the patient was in a somnolent state with an elevated body temperature of 38.5°C and tachypnea. Physical examination revealed multiple erosions and bleeding ulcers on the oral mucosa, alongside extensive petechiae, ecchymoses, and a rash on the face, chest, and trunk (Fig. 1). Bilateral lung auscultation detected moist rales. The abdomen was soft with mild tenderness but no rebound tenderness. Initial laboratory investigations demonstrated severe bone marrow suppression (Table 1), with a leukocyte count of 0.07 × 10^9^/L, a platelet count of 2 × 10^9^/L, and a hemoglobin level of 48 g/L. The serum MTX concentration remained critically high at 7.2 μmol/L 2 days after the last dose. Liver function tests showed elevated alanine aminotransferase (147 U/L) and aspartate aminotransferase (191 U/L). Renal function had deteriorated further, with serum creatinine rising to 906 μmol/L and urea to 51.8 mmol/L. Markers of infection were significantly elevated, with procalcitonin at 3.70 ng/mL and interleukin-6 exceeding 5000 pg/mL (Table 2). Serial coagulation and inflammatory markers stayed persistently abnormal. A comprehensive reduction in lymphocyte subsets indicated severe immunodeficiency (Table 3). Imaging studies, including computed tomography of the chest and abdomen, revealed a lacunar cerebral infarction, along with small pericardial, bilateral pleural, and abdominopelvic effusions, as well as perirenal stranding (Fig. 2).

**Table 1:** Routine blood test results of the patient on admission.

Test name	Result	Reference range
WBC count (x 109/L)	0.07	3.50–9.50
RBC count (x 1012/L)	2.45	4.30–5.80
Hemoglobin (g/L)	48	130–175
Hematocrit %	23.70	40.00–50.00
Mean corpuscular volume (fl)	96.9	82.0–100.0
Mean corpuscular hemoglobin (pg)	32.7	27.0–34.0
Mean corpuscular hemoglobin concentration (g/L)	338	316–354
Platelet count (x109/L)	2	85–303
Platelet distribution width (SD) (fl)	/	10–18
Mean platelet volume (fl)	/	7.6–13.2
Platelet hematocrit (%)	/	0.1–0.5
Neutrophil percentage (%)	/	40.0–75.0
Lymphocyte percentage (%)	/	20.0–50.0
Monocyte percentage (%)	/	3.0–10.0
Eosinophil percentage (%)	/	0.4–8.0
Basophil percentage (%)	/	0.0–1.0
Neutrophil absolute value (x 109/L)	/	1.80–6.30
Lymphocyte absolute value (x 109/L)	/	1.10–3.20
Monocyte absolute value (x 109/L)	/	0.10–0.60
Eosinophil absolute value (x 109/L)	/	0.02–0.52
Basophil absolute value (x 109/L)	/	0.00–0.06

RBC = red blood cell, WBC = white blood cell.

**Table 2:** Biochemical test results of the patient on admission.

Test name	Result	Reference range
Serum methotrexate concentration (μmol/L)	7.2	48 h, ≤ 1.0; 72 h, ≤ 0.1–0.2
Creatinine (μmol/L)	906	58–110
Urea (μmol/L)	51.8	3.2–7.1
AST (u/L)	191	17–59
ALT (u/L)	147	< 50
Lactate dehydrogenase (u/L)	536	120–246
Albumin (g/L)	25	35–50
Interleukin-6 (pg/ml)	> 5000	0–6.6
Procalcitonin (ng/ml)	3.70	-

ALT = alanine aminotransferase, AST = aspartate aminotransferase.

**Table 3:** Results of immune cell count tests of the patient on admission.

Test name	Result	Reference range
Total lymphocytes (cells/u)	290	1752–2708
Total T lymphocytes (CD3+) (cells/u)	146	1185–1901
Helper/inducer T cells (CD4 + T) (cells/u)	115	561–1137
Suppressor/cytotoxic T cells (CD8 + T) (cells/u)	37	464–754
Double positive T cells (CD3 + CD4 + CD8+) (cells/u)	6	0–30
Double negative T cells (CD3 + CD4 − CD8−) (cells/u)	6	0–290
B lymphocytes (CD19 + B) (cells/u)	82	181–324
NK cells (CD3-CD16/56+) (cells/u)	60	175–567
NKT cells (CD3 + CD16/56+) (cells/u)	2	-
Total lymphocytes %	87.10	20.00–50.00
Total T lymphocytes (CD3+) %	50.49	62.6–76.8
Helper/inducer T cells (CD4 + T) %	39.78	30.00–46.00
Suppressor/cytotoxic T cells (CD8 + T) %	12.73	19.20–33.60
Double positive T cells (CD3 + CD4 + CD8+) %	2.02	0–2.00
Double negative T cells (CD3 + CD4 − CD8−) %	2.02	0–12.00
B lymphocytes (CD19 + B) %	28.40	8.50–14.50
NK cells (CD3-CD16/56+)%	20.64	9.50–23.50
NKT cells (CD3 + CD16/56+)%	0.60	-
CD4+/CD8 + ratio	3.1	0.9–3
CD20 + CD19 + co-positive cells	1.000	-

CD = cluster of differentiation, NK = natural killer, NKT = natural killer T-cell.

### 2.4. Diagnostic assessment

This case was diagnosed as severe MTX toxicity caused by impaired drug excretion due to stage 5 CKD. The diagnosis was based on a clear history of MTX use, characteristic mucosal lesions and rash, and persistently elevated blood drug concentrations following administration, followed by severe pancytopenia, immunodeficiency, septic shock, and multiple organ dysfunction syndrome.

### 2.5. Therapeutic intervention

Following admission, MTX was immediately discontinued, and a comprehensive management protocol was initiated. To counteract drug toxicity, leucovorin calcium (15 mg every 6 hours) was administered as a rescue therapy. Continuous renal replacement therapy (hemofiltration-dialysis mode) was implemented to eliminate the accumulated drug and its metabolites and to correct the internal milieu disturbances. In response to the severe bone marrow suppression, supportive treatments including granulocyte colony-stimulating factor and thrombopoietin were administered, alongside transfusions of red blood cells, platelets, fresh frozen plasma, and cryoprecipitate to address anemia and coagulopathy. Given the presence of severe immunodeficiency, high fever, and markedly elevated inflammatory markers suggestive of a secondary infection, empirical antimicrobial therapy with meropenem and caspofungin was commenced. As the patient’s condition continued to deteriorate, with progressive impairment of consciousness, gastrointestinal bleeding, and circulatory and respiratory failure, subsequent interventions included orotracheal intubation and invasive mechanical ventilation.

### 2.6. Follow-up and outcomes

Despite the aggressive and comprehensive therapeutic measures undertaken, the patient’s clinical status showed no improvement. A follow-up serum MTX level measured 6 days after the last dose remained significantly elevated at 2.00 μmol/L, indicating persistent drug accumulation. The patient ultimately succumbed to septic shock, circulatory failure, and consequent multiple organ failure.

## 3. Discussion

According to the 2025 European Alliance of Associations for Rheumatology Guidelines for RA and the 2024 Kidney Disease: Improving Global Outcomes Guidelines for CKD,^[8,9]^ a comprehensive renal function assessment must be performed before initiating MTX therapy, as impaired renal function is a key risk factor for severe adverse reactions to this drug. Unlike high-dose MTX chemotherapy regimens used in oncology, routine low-dose MTX in rheumatology is contraindicated in patients with stage 5 ESRD. A case report has described a patient with stage 3b CKD and psoriasis who developed MTX-induced pancytopenia and mucosal damage following treatment, which improved after symptomatic and supportive therapy,^[10]^ suggesting that different renal function classifications are associated with significant differences in treatment outcomes. In ESRD, renal excretory function is nearly lost, making patients highly susceptible to drug accumulation. This is evidenced by the fact that the serum drug concentration was 7.2 μmol/L 48 hours after administration and remained as high as 2.00 μmol/L on the sixth day of treatment. Numerous previous studies have confirmed that high-dose MTX chemotherapy can cause acute liver injury.^[11]^ However, the clinical course of this case suggests that even a routine low dose of 10 mg per week for rheumatology can induce fatal multiorgan injury in patients with ESRD. This indicates that baseline renal function, rather than the administered dose, is the key factor determining the severity of MTX toxicity.^[12]^

The continuous accumulation of MTX can trigger a cascade of damage: the drug inhibits proliferating cells in the bone marrow and gastrointestinal tract, leading to severe pancytopenia, disruption of the mucosal barrier, and secondary systemic infections.^[13]^ Subsequent sepsis and elevated cytokine levels can induce widespread endothelial damage and multiorgan involvement. Current research suggests that MTX-related kidney injury further impairs residual renal excretory capacity, delays drug clearance, and accelerates disease progression.^[14]^ In summary, following MTX toxicity in patients with ESRD, the condition tends to manifest as progressive multisystem damage.

The patient in this case experienced MTX accumulation, bone marrow suppression, and septic shock, which progressively led to multiple organ failure. The overall clinical course is consistent with previous literature reports.^[15]^ The ideal management strategy for severe MTX toxicity involves early administration of glucarpidase, a specific antidote to reverse delayed MTX clearance, combined with calcium folinate, supplemented by high-flux dialysis to accelerate drug clearance when necessary.^[16,17]^ However, existing studies suggest that once organ damage has occurred, the benefits of conventional rescue measures are significantly limited.^[18]^ In this case, glucarpidase was not available; only calcium folinate combined with blood purification was administered, yet this ultimately failed to halt disease progression, resulting in death.

In conclusion, lessons drawn from this case inform practical prescribing precautions for MTX administration in patients with renal impairment: evaluate renal function prior to every prescription; avoid MTX in patients with ESRD unless compelling clinical indications exist and medication is administered under specialist oversight; fully document all concurrent medications taken by the patient; arrange periodic laboratory monitoring of complete blood count, hepatic and renal function throughout treatment; educate patients to promptly seek medical care upon developing oral ulceration, rash, vomiting, or fever. Pre-prescription risk stratification is indispensable, as severe MTX toxicity carries a grave prognosis even with maximized standard rescue interventions.

This report has inherent limitations due to its retrospective, single-case study design. As a retrospective single-case report, this study can only infer a clinical association between MTX and the patient’s death, but cannot establish a definitive causal relationship. Incomplete prescription records from other hospitals and the lack of continuous baseline laboratory test results limited in-depth analysis. Confounding factors, such as unrecorded concomitant medications and occult subclinical infections, cannot be ruled out. Therefore, the conclusions of this study cannot be directly generalized to similar patient populations. Prospective studies are still needed to determine safe dosing thresholds for patients with end-stage CKD, identify early markers of toxicity, and explore new detoxification strategies.

## Author contributions

**Conceptualization:** Xu Huang.

**Data curation:** Xu Huang.

**Formal analysis:** Xu Huang.

**Software:** Qian He.

**Validation:** Qian He.

**Visualization:** Qian He, Xu Huang.

**Writing – original draft:** Qian He, Xu Huang.

**Writing – review & editing:** Qian He.

## References

[1] Krasselt M. Methotrexate - Safe backbone for the treatment of rheumatoid arthritis. Curr Rheumatol Rev. 2025;21:169–81. 38982927 10.2174/0115733971317122240626053727

[2] Ouellette S Shah R Razi S Ashforth G Wassef C. Fatal low-dose methotrexate toxicity: a case report and literature review. Dermatol Ther. 2022;35:e15945. 36259229 10.1111/dth.15945

[3] Kystaubayeva Z Zhakupbekova M Mukhamedjanova A Nurpeissova R. Hematological side effects of methotrexate in rheumatoid arthritis: a systematic review [published online ahead of print May 15, 2026]. Curr Rheumatol Rev. doi: 10.2174/0115733971454973260424092936. 10.2174/0115733971454973260424092936 42163751

[4] Kanderi T Gomez JC Puthenpura MM. Pancytopenia as a complication of low-dose methotrexate in a septuagenarian: a rare presentation. Cureus. 2020;12:e8492. 32656010 10.7759/cureus.8492 PMC7343297

[5] Javed K Wijeratne R Bandaru SK. The unseen danger of methotrexate toxicity. J Community Hosp Intern Med Perspect. 2023;13:39–42. 10.55729/2000-9666.1255 PMC11000842 38596556

[6] Ratanapokasatit Y Jurairattanaporn N. Methotrexate-induced leukocytoclastic vasculitis: a case report and literature review. Clin Cosmet Investig Dermatol. 2025;18:2005–12. 10.2147/CCID.S528734 PMC12393089 40893823

[7] Ebisawa K Iwashita T Uchiyama K Kitayama Y Takeuchi T. Epstein-Barr virus-positive mucocutaneous ulcer resulting in severe methotrexate intoxication: a case report. J Med Case Rep. 2024;18:409. 39210427 10.1186/s13256-024-04730-w PMC11363652

[8] Smolen JS Edwards CJ Konzett V. EULAR recommendations for the management of rheumatoid arthritis with synthetic and biologic disease-modifying antirheumatic drugs: 2025 update. Ann Rheum Dis. 2026;85:991–1009. 41826212 10.1016/j.ard.2026.01.023

[9] Kidney Disease: Improving Global Outcomes (KDIGO) CKD Work Group. KDIGO 2024 clinical practice guideline for the evaluation and management of chronic kidney disease. Kidney Int. 2024;105(4 Suppl):S117–314. 38490803 10.1016/j.kint.2023.10.018

[10] Surapaneni D Dasi SC Sam N M J. Methotrexate toxicity-induced pancytopenia and mucocutaneous ulcerations in psoriasis. Cureus. 2024;16:e66222. 39238709 10.7759/cureus.66222 PMC11374579

[11] Corbisier T Von Bueren AO Breunis WB. Role of molecular adsorbent recirculating system in methotrexate-induced acute liver failure: a case report and literature review. Front Pediatr. 2024;12:1424919. 39220154 10.3389/fped.2024.1424919 PMC11363709

[12] Zhang L Li J Wang X. Low-dose methotrexate adverse reaction risk in renal impairment: pharmacovigilance and physiological pharmacokinetic model assessment. Front Pharmacol. 2025;16:1703557. 41282623 10.3389/fphar.2025.1703557 PMC12631143

[13] Lui SW Lu JW Ho YJ Hsieh T-Y Yeh F-C Liu F-C. IVIG as a promising therapy for methotrexate-induced life-threatening neutropenic enterocolitis in an elderly patient with rheumatoid arthritis: a case report and literature review. In Vivo. 2024;38:511–7. 38148101 10.21873/invivo.13468 PMC10756487

[14] Fahmy MM Habib HA Zeidan EM Jardan YAB Heeba GH. Alogliptin mitigates methotrexate-induced nephrotoxicity in a rat model: antagonizing oxidative stress, inflammation and apoptosis. Int J Mol Sci. 2025;26:9608. 41096869 10.3390/ijms26199608 PMC12524975

[15] Refeno V Rasamimanana NG Abasse BA Ramarokoto MPM Fanomezantsoa MJE Randaoharison PG. Methotrexate-induced toxidermia and pancytopenia in a patient with ectopic pregnancy: a case report. J Med Case Rep. 2021;15:579. 34872594 10.1186/s13256-021-03111-x PMC8650356

[16] Tome ACN Santos KF Lima EQ Ramalho RJ. High-flux hemodialysis as rescue therapy for high-dose methotrexate toxicity: case series and clinical insights. Front Med (Lausanne). 2025;12:1495705. 40160322 10.3389/fmed.2025.1495705 PMC11949902

[17] Bielack SS Soussain C Fox CP. A European consensus recommendation on the management of delayed methotrexate elimination: supportive measures, leucovorin rescue and glucarpidase treatment. J Cancer Res Clin Oncol. 2024;150:441. 39356310 10.1007/s00432-024-05945-6 PMC11446969

[18] Purzycki T Knapp M Patel S. Extracorporeal management of severe methotrexate toxicity and acute kidney injury in the absence of glucarpidase. BMJ Case Rep. 2025;18:e267929. 10.1136/bcr-2025-267929 PMC12712783 41397779

